# Dietary hyperoxaluria is not reduced by treatment with lactic acid bacteria

**DOI:** 10.1186/1479-5876-11-306

**Published:** 2013-12-12

**Authors:** Roswitha Siener, Diana J Bade, Albrecht Hesse, Bernd Hoppe

**Affiliations:** 1 Department of Urology, University Stone Centre, University of Bonn, Sigmund-Freud-Straße 25, 53105 Bonn, Germany; 2 Department of Pediatrics, Division of Pediatric Nephrology, University of Bonn, Adenauerallee 119, 53113 Bonn, Germany

**Keywords:** Lactic acid bacteria, Lactobacillus, Oxalate-degrading bacteria, Probiotics, Oxalate-rich diet, Dietary hyperoxaluria, Calcium oxalate stone formation

## Abstract

**Background:**

Secondary hyperoxaluria either based on increased intestinal absorption of oxalate (enteric), or high oxalate intake (dietary), is a major risk factor of calcium oxalate urolithiasis. Oxalate-degrading bacteria might have beneficial effects on urinary oxalate excretion resulting from decreased intestinal oxalate concentration and absorption.

**Methods:**

Twenty healthy subjects were studied initially while consuming a diet normal in oxalate. Study participants were then placed on a controlled oxalate-rich diet for a period of 6 weeks. Starting with week 2 of the oxalate-rich diet, participants received 2.6 g/day of a lactic acid bacteria preparation for 5 weeks. Finally, subjects were examined 4 weeks after treatment while consuming again a normal-oxalate diet. Participants provided weekly 24-hour urine specimens. Analyses of blood samples were performed before and at the end of treatment.

**Results:**

Urinary oxalate excretion increased significantly from 0.354 ± 0.097 at baseline to 0.542 ± 0.163 mmol/24 h under the oxalate-rich diet and remained elevated until the end of treatment, as did relative supersaturation of calcium oxalate. Plasma oxalate concentration was significantly higher after 5 weeks of treatment compared to baseline. Four weeks after treatment, urinary oxalate excretion and relative supersaturation of calcium oxalate fell to reach initial values.

**Conclusions:**

Persistent dietary hyperoxaluria and increased plasma oxalate concentration can already be induced in healthy subjects without disorders of oxalate metabolism. The study preparation neither reduced urinary oxalate excretion nor plasma oxalate concentration. The preparation may be altered to select for lactic acid bacteria strains with the highest oxalate-degrading activity.

## Background

Calcium oxalate is the major constituent of about 75% of all urinary stones [1]. Secondary hyperoxaluria either based on intestinal hyperabsorption of oxalate, i.e. enteric hyperoxaluria, or high intake of oxalate, i.e. dietary hyperoxaluria, is considered a primary risk factor in the pathogenesis of calcium oxalate urolithiasis. The impact of dietary oxalate in the development of calcium oxalate stone formation is unclear. It has been suggested that dietary oxalate contributes up to 50% of urinary oxalate excretion [2]. Estimates of normal dietary oxalate intake are in the range of 50 to 200 mg daily, but may exceed 1000 mg per day if oxalate-rich foods are ingested [3-5]. Analysis of the oxalate content of different foods by reliable methods revealed a considerable number of foods with high oxalate concentrations, e.g. spinach, rhubarb, sorrel, sesame, carambola, almonds or wheat bran [4,6,7]. The oxalate content of foods may vary according to growth conditions and preparation methods [6,8]. However, a study in healthy subjects revealed no significant increase in the relative urinary supersaturation of calcium oxalate under a vegetarian diet despite an increased oxalate intake [9]. Moreover, a prospective study reported only a modest positive association between dietary oxalate intake and the risk for incident stone formation [5].

Colonization with oxalate-degrading bacteria in the intestine may contribute to a decrease in urinary oxalate excretion through reduction of intestinal concentration of oxalate available for absorption [10]. Previous studies demonstrated that lactic acid bacteria are able to degrade oxalate *in vitro* and to reduce urinary oxalate excretion in patients with idiopathic as well as enteric hyperoxaluria [11,12]. However, two recent studies in calcium oxalate stone formers with mild hyperoxaluria failed to find a beneficial effect of the same lactic acid bacteria preparation (Oxadrop, VSL Pharmaceuticals, Rome, Italy) on urinary oxalate excretion and calcium oxalate supersaturation [13,14].

Aim of this study was to examine the effect of oral administration of the lactic acid bacteria preparation on dietary hyperoxaluria, plasma oxalate concentration and the relative urinary supersaturation of calcium oxalate in healthy subjects under controlled standardized conditions. Thus, a major challenge for this study was the induction of hyperoxaluria by high dietary intake of oxalate.

## Subjects and methods

### Subjects

A total of 20 healthy subjects, including 10 women and 10 men participated in the study. Inclusion criteria required that participants (a) did not have a medical history of stone formation or overt gastrointestinal diseases, (b) showed no evidence of stone formation or other urinary tract diseases in current renal ultrasound, (c) had normal kidney function and (d) urinary oxalate excretion < 0.5 mmol/24 h prior to the study. Each subject had a normal physical examination and normal findings from multiparameter urine test strips (Combur^9^-Test, Roche Diagnostics GmbH, Mannheim, Germany). The subjects took no medication, including antibiotics, at least 4 weeks prior to and during the study. The study was approved by the ethics committee of the Medical Faculty of the University of Bonn (reference number 098/02). Written informed consent was obtained from each participant before trial onset.

### Study procedure

The study design is summarized in Table 1. Subjects were studied at baseline while consuming a diet normal in oxalate (approximately 100 mg oxalate/d). Diets were individualized to avoid a strict regimen that is not feasible for a long period, but remained constant for oxalate-containing foods for the duration of the study. Study participants were then placed on a controlled, oxalate-rich diet (approximately 600 mg oxalate/d) for a period of six weeks. Therefore, 150 ml of pure rhubarb juice, containing approximately 500 mg oxalate, was consumed in addition to the normal-oxalate baseline diet. Rhubarb juice was chosen because standardization of dietary oxalate intake can easily be achieved. Rhubarb juice was ingested at lunch, a common time for the consumption of oxalate-rich foods. The rhubarb juice was well tolerated. Starting with week 2 of the oxalate-rich diet, the subjects took one packet q.d. (2.6 g/d) of a lactic acid bacteria preparation (Oxadrop, VSL Pharmaceuticals, Rome, Italy) for five weeks. Subjects were instructed to take the preparation at breakfast. Finally, subjects were examined four weeks after treatment while consuming again a diet normal in oxalate as at baseline (approximately 100 mg oxalate/d).

**Table 1:** Study design

**Controlled diet**	**Normal-oxalate diet**	**Oxalate-rich diet**						**Normal-oxalate diet**
**Phase**	**Baseline**	**Control**	**Intervention**					**Follow-up**
**Week**	**Week 0**	**Week 1**	**Week 2**	**Week 3**	**Week 4**	**Week 5**	**Week 6**	**Week 10**
Lactic acid bacteria			X	X	X	X	X	
24 h-urine	X	X	X	X	X	X	X	X
Blood sample	X						X	

The composition of the diet was assessed by a 7-day weighed dietary record. The subjects provided a detailed description of types and weighed amounts of all food items consumed. Subjects were asked to maintain a similar diet during the study. Dietary records were used by study participants to replicate dietary intake and collected by the investigator to verify oxalate intake through analysis of the oxalate content of foods. A blood sample was obtained at baseline and at the end of treatment with the study preparation (week 6). In addition, 24-hour urines were weekly collected during the study. Renal ultrasound examinations were performed at baseline and after the treatment period to exclude either stone formation or nephrocalcinosis.

### Study preparation

Each gram of the lactic acid bacteria mixture (Oxadrop, VSL Pharmaceuticals, Rome, Italy) contained 3.6 × 10^11^ CfU (*Lactobacillus acidophilus*, *Bifidobacterium infantis*, *Streptococcus thermophilus* and *Lactobacillus brevis*). The study preparation was kept refrigerated at 4°C and administered at breakfast.

### Dietary oxalate concentration

To assure dietary oxalate intake, the oxalate contents of foods were measured. For the determination of total oxalate concentration, oxalate was extracted with 2 N hydrochloric acid from homogenized samples. Analysis of filtrates was performed by the HPLC-enzyme-reactor method [15]. Oxalate was separated from matrix substances by an anion exchange column (AS4A-DIONEX, ThermoFisher Scientific, Waltham, Massachusetts). The mobile phase consisted of 2 g/l EDTA solution adjusted to pH 5.0 with 0.3 mol/l NaOH. The enzyme reactor contained 5 units of immobilized oxalate oxidase (oxalate oxidase: Sigma Diagnostics, St. Louis, USA) (carrier: VA Epoxy Biosynth, Riedel-de-Häen, Seelze, Germany) which oxidized oxalate to carbon dioxide and hydrogen peroxide. Resulting hydrogen peroxide was analyzed by amperometric detection.

### Plasma oxalate concentration and serum profile

Plasma oxalate concentration was measured using an HPLC enzyme reactor method [16]. Blood samples were obtained in lithium heparin tubes and immediately centrifuged at 1600 rpm (1000 × g) for 10 min at 4°C. Ultrafiltration was carried out in a Centrisart-I-tube (cut off 20,000 D, Sartorius, Germany) at 3900 rpm (2500 × g) for 50 min. at 4°C. To remove proteins and macromolecules, 0.5 mL plasma was added to 0.5 mL distilled water in the outer tube. To prevent in vitro generation of oxalate, 12.5 μL of 1 N hydrochloric acid was added to the inner tube. The yielded ultrafiltrate was analyzed for oxalate concentration using HPLC enzyme reactor. Analysis of serum parameters was performed by routine methods.

### 24 h urinary excretion profiles

Urine volume, pH (potentiometry) and concentrations of creatinine (Jaffé reaction), sodium, potassium and chloride (indirect ion selective electrode), calcium (cresolphthalein complex), magnesium (methylthymol blue), inorganic sulfate (nephelometry), inorganic phosphate (phosphate molybdate reaction), ammonium (ion selective electrode), citrate (enzymatically, citrate lyase), uric acid (enzymatically, uricase), and oxalate (enzymatically, oxalate oxidase) were measured. Laboratory quality certification was available for each parameter. The relative supersaturation of calcium oxalate (RS CaOx) was determined using EQUIL2 [17].

### Statistics

Comparisons between groups were performed using the Mann–Whitney U-test for non-parametric unpaired data. Differences between values at baseline, control, intervention and follow-up were evaluated by the two-tailed Wilcoxon matched pairs signed rank test. Data are presented as means ± standard deviation. All reported P values are two-sided. Differences were considered significant at p < 0.05. Statistical analysis was performed using IBM Statistical Package for the Social Sciences version 21.0 (SPSS Inc., Chicago, IL, USA).

## Results

Baseline characteristics of the study participants are listed in Table 2. There were 20 healthy subjects, 10 men and 10 women, of a mean age of 29 ± 9 years (range 22 – 62 years) and a mean body mass index (BMI) of 23 ± 2 kg/m^2^ (range 20 – 27 kg/m^2^). Mean age and BMI did not differ between men and women.

**Table 2:** Characteristics of healthy subjects (mean ± standard deviation)

	**Total**	**Men**	**Women**	**P**
	**(n = 20)**	**(n = 10)**	**(n = 10)**	**M vs. W**
Age (years)	29.1 ± 9.3	29.6 ± 12.3	28.6 ± 5.3	0.147
Height (cm)	176 ± 10	183 ± 8	169 ± 5	
Weight (kg)	70 ± 12	78 ± 11	63 ± 7	
BMI (kg/m^2^)	22.6 ± 2.0	23.2 ± 2.0	22.0 ± 1.9	0.199

Table 3 shows urinary parameters. Urinary oxalate excretion increased significantly from 0.354 ± 0.097 at baseline on the normal-oxalate diet to 0.542 ± 0.163 mmol/24 h after 1 week under the controlled oxalate-rich diet, as did relative supersaturation of calcium oxalate. All other urinary parameters, except urinary chloride excretion, did not change. No consistent change in any urinary parameter occurred during administration of the study drug compared to control. Changes were observed in exceptional cases regarding urinary potassium (week 2), and citrate excretion (week 6), possibly due to small variations in dietary composition. The lactic acid bacteria preparation was well tolerated. No subject had any abdominal discomfort or did complain about other adverse events. During follow-up (week 10) under controlled normal-oxalate diet, urinary oxalate excretion and relative supersaturation of calcium oxalate fell to reach initial values.

**Table 3:** Urinary parameters at baseline, before (control), during (intervention) and after administration (follow-up) of the study preparation (n = 20; mean ± standard deviation)

	**Baseline**	**Control**	**Intervention**		**Follow-up**	**P**	**P**	**P**	**P**
**Diet**	**Normal-oxalate**	**Oxalate-rich**	**Oxalate-rich**	**Oxalate-rich**	**Normal-oxalate**				
**Week**	**Week 0**	**Week 1**	**Week 2**	**Week 6**	**Week 10**	**Wk 0 vs. 1**	**Wk 1 vs. 2**	**Wk 1 vs. 6**	**Wk 0 vs. 10**
Volume (mL/24 h)	2,379 ± 1,063	2,396 ± 858	2,322 ± 997	2,534 ±1,311	2,433 ± 1,194	0.888	0.550	0.526	0.765
pH	6.38 ± 0.37	6.36 ± 0.31	6.39 ± 0.38	6.39 ± 0.41	6.39 ± 0.21	0.365	0.823	0.667	0.926
Sodium (mmol/24 h)	157 ± 74	182 ± 51	155 ± 65	173 ± 58	179 ± 74	0.100	0.107	0.398	0.204
Potassium (mmol/24 h)	65 ± 29	71 ± 19	82 ± 24	77 ± 28	74 ± 23	0.287	0.044	0.190	0.232
Calcium (mmol/24 h)	4.02 ± 1.83	4.11 ± 1.70	3.85 ± 1.54	4.09 ± 1.51	4.47 ± 2.16	0.881	0.411	0.808	0.334
Magnesium (mmol/24 h)	5.14 ± 2.06	5.18 ± 1.95	4.68 ± 1.75	4.99 ± 1.87	5.56 ± 2.73	0.970	0.262	0.881	0.351
Ammonium (mmol/24 h)	28.1 ± 9.1	26.6 ± 7.2	28.0 ± 8.3	29.8 ± 9.4	30.0 ± 11.8	0.351	0.526	0.218	0.940
Chloride (mmol/24 h)	147 ± 71	178 ± 48	161 ± 68	158 ± 53	182 ± 82	0.026	0.391	0.070	0.052
Phosphate (mmol/24 h)	26.9 ± 8.6	28.2 ± 7.5	28.8 ± 7.7	31.7 ± 10.5	27.4 ± 9.8	0.478	0.681	0.135	0.881
Sulphate (mmol/24 h)	20.5 ± 6.5	20.7 ± 6.7	22.2 ± 5.9	23.4 ± 5.7	20.6 ± 5.2	0.881	0.218	0.161	0.852
Creatinine (mmol/24 h)	12.43 ± 3.50	13.46 ± 4.87	13.19 ± 4.28	13.27 ± 4.22	12.53 ± 3.97	0.232	0.563	0.279	0.940
Uric acid (mmol/24 h)	3.10 ± 0.90	3.27 ± 1.01	3.16 ± 0.74	3.35 ± 0.73	3.16 ± 0.89	0.305	0.681	0.370	0.695
Oxalate (mmol/24 h)	0.354 ± 0.097	0.542 ± 0.163	0.563 ± 0.183	0.638 ±0.197	0.371 ± 0.108	< 0.001	0.563	0.052	0.455
Citrate (mmol/24 h)	3.662 ± 1.567	3.553 ± 1.123	3.640 ± 1.517	3.969 ± 1.461	3.444 ± 1.233	0.526	0.970	0.021	0.279
RS CaOx	3.211 ± 1.672	4.398 ± 2.317	4.867 ± 2.921	5.111 ± 2.973	3.647 ± 2.151	0.004	0.550	0.108	0.332

Blood parameters are listed in Table 4. Plasma oxalate concentration was significantly higher under the oxalate-rich diet during administration of the lactic acid bacteria preparation (week 6) compared to baseline. All other parameters did not differ significantly. The statistically significant difference in serum cholesterol concentration between intervention and baseline is not considered clinically relevant.

**Table 4:** Blood parameters at baseline (week 0) and end of intervention (week 6) (n = 20; mean ± standard deviation)

	**Baseline**	**Intervention**	
**Week**	**Week 0**	**Week 6**	**P**
Sodium (mmol/L)	139 ± 2	139 ± 2	0.861
Potassium (mmol/L)	4.48 ± 0.40	4.44 ± 0.45	0.598
Calcium (mmol/L)	2.36 ± 0.07	2.35 ± 0.12	0.587
Chloride (mmol/L)	103 ± 2	103 ± 3	0.759
Magnesium (mmol/L)	0.82 ± 0.07	0.81 ± 0.06	0.079
Creatinine (mg/dL)	0.96 ± 0.15	0.95 ± 0.13	0.513
Urea (mg/dL)	26 ± 6	26 ± 5	0.779
Uric acid (mg/dL)	4.7 ± 1.0	4.7 ± 0.9	0.276
Triglycerides (mg/dL)	82 ± 38	89 ± 36	0.184
Cholesterol (mg/dL)	192 ± 26	185 ± 27	0.046
HDL (mg/dL)	67 ± 16	64 ± 15	0.354
LDL (mg/dL)	109 ± 26	103 ± 24	0.056
Leucocytes (G/L)	5.78 ± 1.60	5.87 ± 1.42	0.672
Erythrocytes (T/L)	5.0 ± 0.3	4.94 ± 0.32	0.599
Hemoglobin (g/dL)	14.9 ± 1.1	14.8 ± 1.0	0.615
Hematokrit (%)	44 ± 3	44 ± 3	0.524
Thrombocytes (G/L)	223 ± 48	235 ± 48	0.077
Oxalate (μmol/L)	1.19 ± 0.80	2.85 ± 2.38	0.002

## Discussion

The results of our study clearly reveal the role of dietary oxalate as a major determinant of urinary oxalate excretion. The present data demonstrate that an oxalate-rich diet can induce persistent hyperoxaluria already in healthy subjects without disturbances of oxalate metabolism or gastrointestinal diseases. The daily consumption of an oxalate-rich diet with an oxalate content of approximately 600 mg resulted in a significant increase in urinary oxalate excretion from 0.354 ± 0.097 at baseline under the normal-oxalate diet to 0.542 ± 0.163 mmol/24 h during control by 0.188 mmol/24 h (> 50%), corresponding to 35% of total urinary oxalate excretion. Taking into account that an additional proportion of urinary oxalate comes from the basal diet normal in oxalate, a higher percentage of urinary oxalate excretion may derive from the diet than has been previously assumed [18]. Accordingly, a study by Holmes et al. revealed that dietary oxalate may contribute up to 50% of urinary oxalate excretion in healthy individuals [2].

Our results agree with findings of Hess et al., who reported that urinary oxalate excretion increased to only 0.780 mmol/24 h following the ingestion of a diet rich in oxalate and normal in calcium (2220 mg oxalate and 1211 mg calcium per day) [19]. In the present study, the ingestion of 500 mg of oxalate per day resulted in the absorption of only 3.4% of the additional food derived oxalate. The suppressed absorption of oxalate that is apparent at high oxalate intake is compatible with results of Holmes et al., who calculated that for an intake of 50, 180 and 250 mg oxalate per 2500 kcal/day, the increase in urinary oxalate excretion corresponds to the absorption of 15.1, 6.6 and 5.8%, respectively, of the dietary oxalate [2]. Our results suggest that there is a limit in oxalate absorption, as urinary oxalate does not continuously rise even if dietary oxalate is further increased.

Urinary oxalate concentration plays a crucial role in determining the risk of calcium oxalate stone formation. Because the oxalate/calcium molar ratio in urine is normally 1:10, even small variations in urinary oxalate concentration exert much greater changes in relative supersaturation of calcium oxalate, crystallization and the risk of stone formation than comparable variations in urinary calcium concentration. Thus, the relative supersaturation of calcium oxalate increased significantly under the oxalate-rich diet.

Previous studies suggested that lactic acid bacteria could lower urinary oxalate excretion. A study conducted by Campieri et al. showed that a variety of lactic acid bacteria are able to degrade oxalate *in vitro*, at least to a modest degree [11]*.* The mixture of bacterial strains in Oxadrop were also selected for their ability to degrade oxalate *in vitro*[11]. However, the administration of the lactic acid bacteria preparation in this study in healthy subjects did not affect urinary oxalate excretion. At least two mechanisms are ascribed to the presence of lactic acid bacteria that use oxalate in their metabolism: decrease in intestinal oxalate concentration by degradation of oxalate and decrease in oxalate absorption. Because urinary oxalate excretion did not vary during intervention with the study preparation under the controlled oxalate-rich diet, it is unlikely that the lactic acid bacteria preparation decreased intestinal oxalate concentration and/or absorption. Although a previous small study in six patients with idiopathic calcium oxalate urolithiasis and mild hyperoxaluria as well as a study in ten calcium oxalate stone formers with enteric hyperoxaluria found that lactic acid bacteria may reduce urinary oxalate excretion [11,12], two recent studies in calcium oxalate stone formers with mild hyperoxaluria under controlled conditions were unable to find a beneficial effect of Oxadrop on urinary oxalate excretion and calcium oxalate supersaturation [13,14]. Importantly, the dosage used in these trials was similar to that used in the present study, which was 9.4 × 10^11^ bacteria.

On the contrary to our results, two studies conducted on the lactic acid preparation VSL#3 (Sigma Tau Pharmaceuticals, Gaithersburg, USA), which is similar but not identical in its lactic acid bacteria content and composition to Oxadrop, revealed a significant fall in urinary oxalate excretion and estimated oxalate absorption in healthy subjects [20,21]. However, major study limitations should be considered. Whereas the first study reported an extremely high mean baseline absorption of 31% in healthy subjects, which rather seems to be characteristic for patients with enteric hyperoxaluria secondary to intestinal diseases and/or resection [20], in the second study, intestinal oxalate absorption was assessed only at the 6-hour post-oxalate ingestion period [21]. Moreover, in both studies urinary oxalate excretion was not assessed for the complete 24-hour post-ingestion period.

Since previous studies of Oxadrop also failed to demonstrate an effect [13,14], the preparation may be altered to select specifically for oxalate-degrading strains of lactic acid bacteria. A study in 60 *Lactobacillus* strains belonging to 12 species found a high variability in the oxalate-degrading capacity in the different species [22]. Strains of *Lactobacillus acidophilus* showed the highest oxalate-degrading activity, whereas *Lactobacillus brevis* exhibited no degrading activity. Magwira et al. suggested that *Lactobacillus* and *Bifidobacterium* spp. rather than *Oxalobacter formigenes* may protect black South Africans against calcium oxalate stone disease due to the increased diversity and abundance of these oxalate-degrading strains and low abundance of *Oxalobacter formigenes* in this group [23].

A limitation of our study regarding the presumed lesser ability of the lactic acid bacteria preparation compared with *Oxalobacter formigenes* to directly metabolize oxalate is the possibility that the high oxalate diet has overcome the ability of the preparation to demonstrate an effect. It is unknown whether Oxadrop might have an effect in the current study population if the subjects had been studied on a normal-oxalate diet. However, the administration of the oxalate-rich diet was required to induce dietary hyperoxaluria and to achieve adequate intestinal growth and activity of the oxalate-degrading lactic acid bacteria in Oxadrop, as has been shown in animal studies with *Oxalobacter formigenes*[24]*.* Moreover, studies, confirming the ability of the study preparation to survive passage through extremely acidic environments, such as the human stomach, and to be present in the location where oxalate absorption occurs, are lacking.

To our knowledge, studies examining the effect of administration of lactic acid bacteria on plasma oxalate concentrations are lacking. The plasma oxalate concentration in healthy subjects ranges between 1 and 16 μmol/L and are found to be age-related with lower concentrations in younger subjects [16]. Plasma oxalate concentrations are significantly higher in patients with primary hyperoxaluria, especially, when the glomerular filtration rate declines and chronic kidney failure becomes obvious [25]. In the present study, the ingestion of the oxalate-rich diet resulted in an increase in plasma oxalate concentration already in healthy subjects. Because lactic acid bacteria preparation did not affect urinary oxalate excretion, and hence intestinal oxalate absorption, it can be assumed that a high percentage of plasma oxalate is derived from the diet.

## Conclusions

The current study suggests that persistent dietary hyperoxaluria can already be induced in healthy subjects without disturbances of oxalate metabolism simply by ingestion of foods with high oxalate content. The lactic acid bacteria preparation used in our study did not reduce urinary oxalate excretion. Interestingly and surprisingly, plasma oxalate concentration increased under the controlled oxalate-rich diet irrespective of the administration of the lactic acid bacteria preparation. Despite evidence in the literature that lactic acid bacteria may play a role in the therapy of calcium oxalate stone formers with idiopathic or enteric hyperoxaluria, we were not able to show effectiveness of these bacteria in the treatment of dietary hyperoxaluria in healthy subjects. Therefore, the preparation may be altered to select for lactic acid bacteria strains with the highest oxalate-degrading activity.

## Competing interests

Bernd Hoppe is a consultant to Oxthera Corp and Allena Pharmaceuticals. The Oxadrop preparation and financial support was provided by VSL Pharmaceuticals, Gaithersburg, FL, USA.

## Authors’ contribution

DJB, AH and BH designed the study; DJB conducted the study; DJB, BH and RS analysed data; RS wrote the paper. All authors read and approved the final manuscript.
